# Antiretroviral Treatment Adherence Rate and Associated Factors among People Living with HIV in Dubti Hospital, Afar Regional State, East Ethiopia

**DOI:** 10.1155/2015/187360

**Published:** 2015-04-09

**Authors:** Bekele Belayihun, Rahma Negus

**Affiliations:** ^1^Ethiopian Public Health Association, P.O. Box 7117, Ethiopia; ^2^Afar Regional Health Bureau, Ethiopia

## Abstract

*Introduction*. Antiretroviral Therapy has transformed HIV infection into a chronic manageable disease; it requires near perfect adherence rates (as high as 95%). In this study, we assessed antiretroviral treatment adherence rate and associated factors among people living with HIV in Dubti Hospital. *Methods*. A retrospective cross-sectional study design was conducted within February 1–30, 2014. All HIV-infected patients above the age of 18 years who took first line Antiretroviral Therapy were eligible for inclusion of the study. Adherence Scale was used for labeling patients as adherent or nonadherent. All HIV-infected patients record data were collected from the medical records, entered, and analyzed using Epi Info 7 and SPSS Version 20. Multivariable analysis was used to identify the relative effect of explanatory variables on low adherence rate. *Results*. A total of 370 patients aged 18 years and above, who started ART, were included in this study. The self-reported adherence rate of the patient on ART was 81.1%. Independent predictors of adherence were treatment duration. *Conclusion*. Adherence rate was associated with time to ART. That is, the longer they were on ART, the lesser they adhered.

## 1. Introduction

In an effort to improve the quality of life among HIV-infected people, multiple strategies including treatment of patients with Antiretroviral Therapy have been implemented worldwide. Antiretroviral medications for HIV/AIDS are among the most efficacious and the most life transforming of therapeutic innovations of recent years. Antiretroviral Therapy (ART) changes a uniformly fatal disease to a manageable chronic illness with proper use and good adherence level. Antiretroviral Therapy requires near perfect adherence rates (as high as 95%) [1–3]. Failure to observe this adherence threshold leads to treatment failure, higher mortality rate, lower rate of increasing CD4 cell count and undetectable viral load, lower therapeutic success, disease progression and emergence of drug resistant HIV/IADS strains, and increase in hospital days [4].

Unfortunately, maintaining adequate levels of adherence to antiretroviral medications has proved challenging not only for persons living with HIV, but also for healthcare providers because a failing regime as a result of poor adherence will lead to increased opportunistic infections, increased hospitalization, and outpatient visits and thus increased work load [2, 4]. Suboptimal adherence with resultant treatment failure is still common [5]. Inadequate adherence to treatment is associated with detectable viral loads, declining CD4 counts, disease progression, episodes of opportunistic infections, and poorer health outcomes. Nonadherence may eventually damage the dramatic improvements in HIV-related health parameters [6].

Adherence to medication is a dynamic behavior affected by factors related to treatment regimen complexity, patient-related variables, patient-healthcare provider relationships, and the quality of healthcare services [7]. Patient adherence to ART is influenced by regimen-related factors such as pill burden, frequency of dosing, adverse drug reactions (ADRs) and fluid, and dietary restrictions [8]. Similarly, patient-related factors such as lack of transport, shortage of food, use of traditional medicine, alcohol abuse, depression, stigma and discrimination, and lack of social support undermine adherence [9]. Further, a poor patient-healthcare provider relationship and low quality services, such as lack of confidentiality and privacy and drug stock-outs, can hamper adherence with ART [10], despite the fact that assessing the level of adherence to ART and determinant factors is crucial for further improvement of ART adherence.

Ethiopia is one of the seriously affected countries in sub-Saharan Africa, with more than 1.3 million people living with HIV [6]. In Ethiopia the adult prevalence of HIV was estimated to be 1.5% in 2011 [11]. ART has provided dramatic reductions in hospitalization and mortality rates. It has also increased the quality of life for many individuals living with HIV [12]. Although some studies demonstrate that the incidence of HIV-related wasting syndrome has also declined in the ART era, data from the Nutrition for Healthy Living (NFHL) cohort showed that weight loss and wasting are still common in HIV-infected people and that even a 5% weight loss in 6 months markedly increases the risk of death [13]. Few studies have evaluated adherence rate of ART patients in different areas of the country. Two studies in Ethiopia reported 81.2% and 82.8% adherence to more than 95% of doses [14, 15]. But adherence rate of ART and associated factors among people living with HIV are not well understood in our region, because Afar region is Erta Ale active volcanic movement, Great Rift Valley, and it has the lowest point in Ethiopia and also in Africa which is a different topographical and living culture compared to other regions in Ethiopia. The population in Afar is pasture; they were affected by wasting and poor nutrition. Therefore the main aim of this study was to assess the adherence including factor associated with adherence among ART patients in Dubti Hospital, Afar region, East Ethiopia.

## 2. Methods 

### 2.1. Study Design, Period, and Setting

A hospital based retrospective cross-sectional study design was carried out within February 1–30, 2014, at Dubti Hospital in Afar region using secondary data source. Afar Regional State is one of the nine regional states, administratively divided into five zones and 32 woredas, and Semera is the administrative capital of the region. The Region is located bordering Eritrea in the North, Djibouti in the East, Tigray and Amhara in the West, Oromia and Somali regional state South and South East, respectively. The Region lies in the Great Rift Valley of East Africa about 120 meters below sea level around the Dallol depression. It has an area of 97,256 km^2^ with an estimated 1,411,092 population according to the 2011 census and projection. The Region is generally characterized by desert climatic condition with stony and sandy crust lands and harsh climate for most of the year with annual temperature between 26 and 52 degrees Celsius in the Dallol Woreda. Afar ethnicity is the dominant native of the region particularly in the rural area, with the Muslim dominant religion followers. Hospital gives service for thousands of HIV/AIDS patients since 2005 from rural and urban areas. There were 2350 HIV/AIDS patients on follow-up among which 1059 started ART treatment and were on follow-up.

### 2.2. Study Population and Sample Procedures

The study population consists of all HIV-infected patients aged 18 and above who were seen at Dubti Hospital HIV Clinic, Afar region. The study participants were any people living with HIV who had been on ART in the hospital within February 1–30, 2014, and who have complete registration; intake and follow-up forms were included. Patients who transferred out and lost to follow-up (drop, lost), women who were pregnant, and lactating mothers in WHO stage I or II within February 1–30, 2014, were excluded from the study. The study subjects were randomly selected based on the inclusion criteria. Profiles of all patients on ART during the study period were evaluated. The sample size was calculated using single population proportion formula with the assumption of proportion of Antiretroviral Treatment adherence of (*P* = 0.73) [16], 5% precision, and at 95% confidence interval. Finally add 10% of the calculated value to compensate for possible missing records. Then, simple random sampling technique was employed separately to select 370 samples using computer-generated random number table.

### 2.3. Data Collection

Once the desired patient medical records were identified from the eligible medical records, the required information from the patient medical records was collected using data abstraction form developed to capture basic information about clinical, laboratory, and demographic record. Data abstraction format was developed from ART entry and follow-up form being used in the ART clinic of the Dubit Hospital and Ministry of Health. Pretest of the data abstraction forms was done to get concrete insights into the study record, how data's abstracted from medical records, and were finalized data abstraction form. An input that was obtained from the pretest was incorporated into the data abstraction forms. A total of two days of training were given for data collectors and supervisors. Data quality was censured by using pretested data abstraction form and close supervision by checking the collected data daily for completeness. All the completed data were examined again for completeness and consistency during data entry, storage, and analysis by principal investigator.

### 2.4. Variables

The main outcome measure was self-reporting adherence rates within February 1–30, 2014, on ART follow-up date. The independent variables were sex, age, marital status, educational status, occupation, residency, duration on ART, CD4 count, WHO stage, type of ART drug used, and disclosure status.

### 2.5. Data Analysis

Data was entered and cleaned using Epi Info Version 7. SPSS Version 20 was used for analysis to measure the association of patient's characteristics on adherence rate and calculate adherence rates of the patients. Multivariable logistic regression model was used to identify associated factors of adherence rate using odd ratios with 95% CI. Significant factors in a bivariate analysis with *P* < 0.25 were included in a multivariable logistic regression analysis.

### 2.6. Ethical Consideration

Ethical approval from the institutional review board of the Dubti Hospital was obtained. Permission to conduct the study in follow-up unit was obtained from the medical director's office of the hospital. Names or identification numbers of HIV/AIDS patients were not included in the data abstraction sheet.

## 3. Result

### 3.1. Sociodemographic and Clinical Characteristics

A total of 370 patients aged 18 years and above, who started ART, were included in this study. From the study participants, 210 (56.8%) of them were females and the mean age was 32.9 (SD = 9.1) and also 171 (46.2%) were in age group greater than 30 years. Eighty-two percent (82.2%) of the cohort started ART on the late stage of the disease (stages 3-4). The median CD4 count was 169 cells/*μ*L (IQR = 87.5–261.3 cells/*μ*L). Three hundred and two (81.6%) of the patients had disclosed their serostatus to their partner and other close relatives. the mean treatment duration on ART was 47.7 months (s.d = 26.3 months). The minimum treatment duration was 1 month and the maximum was 104 months. The overall prevalence of self-reported adherence rate was 81.1% (Table 1).

### 3.2. Factors Associated with Self-Reported Adherence Rate

The association of selected sociodemographic, clinical, and other characteristics on adherence status was investigated using both the bivariate and multivariable logistic regression techniques. Accordingly, variables that were significantly associated with self-reported adherence rate in the bivariate analysis were WHO clinical stage, baseline CD4 count, occupation status, place of residence, and treatment duration. Explanatory variables with *P* value up to 0.25 were included in the multivariable logistic regressions. Finally in the multivariable logistic regression analysis showed those patients was longer treatment duration on ART, the lesser they adhered (AOR = 0.14, 95% CI = (0.056–0.33) (Table 2).

## 4. Discussion

Ensuring Antiretroviral Therapy patients' adherence is one of the major challenges to public health in many developing countries. Good adherence to antiretroviral medications is critically important for the success of therapy in patients who are on ART. Proper use of ART suppresses HIV viral replication, consequently slowing down disease progression, improving immunity, and delaying mortality. In our finding, self-reported adherence rate of HIV patients who were on ART was found to be higher than in most developed countries, where rates ranged from 50% to 70% [17, 18]. The findings were in line with other studies in Ethiopia (81.2%, 82.8%) [14, 15]. The level of adherence was comparable with those reported in Harari (87%), Yirgalem (88.7%), and Wolaytasodo (87.5%) [16, 19, 20] but slightly lower, it might be Afar regional settings climatic condition is desert with stony and sandy crust lands and cruel climate. This climatic condition contributes to low nutritional status of the patients compared to Harari, Yirgalem, and Wolaytasodo settings. Low nutritional status (shortage of food) was a major reason for nonadherence to ARTs as these drugs were said to increase appetite [21].

There was association between time of ART treatment and self-reported adherence. Also note that a negative association between time on ART and adherence may also stem from the fact that individuals feel tired after taking their medications over long periods of time and they felt healthy [22]. Similar findings were documented in Ibadan, Nigeria [23], and Ethiopia [24]. Other similar studies done in Africa, in the early therapy; HIV positives adhere more because they experience a dramatic increase in their health status [25]. The adherence rate of ART patients increased with an increase in their educational level. Generally, better educated have access to information and are more likely to make better informed decisions [25]. Verbal instructions to patients who are illiterate seem equally as effective as written instructions which are given to all patients [26]. But in our finding there was no significant association between the adherence rate and the educational level.

In conclusion, this study showed that the self-reported adherence rate of Afar region was higher than in most developed countries but in line with other similar studies in Ethiopia. Time of ART associated with self-reported adherence rate.

There are limitations to our study because of its cross-sectional design and the convenience of the sample used. Being an intended baseline study, we assessed only self-reported adherence to therapy at a single time point. This therefore makes it impossible to evaluate variations in adherence over time for an individual. Self-reporting was used as the only method of measuring adherence. This method has the disadvantages of recall bias and eliciting only socially acceptable responses and hence may overestimate the level of adherence. Hence, the extent of generalizability is limited only to those similar patients.

## Figures and Tables

**Table 1:** The sociodemographic and clinical characteristics of participants.

Variable	Categories	Frequency	Percent (%)
Age	<25	85	23
	25–30	114	30.8
	>30	171	46.2
*Mean and s.d of participant age *			32.9 (SD = 9.1)
Sex	Female	210	56.8
	Male	160	43.2
Residence	Rural	69	15.6
	Urban	301	84.4
Religion	Muslim	258	69.7
	Christian Orthodox	112	30.3
Educational status	No school	173	46.8
	Primary	108	29.2
	Secondary & above	89	24
Occupation	Employed	215	58.1
	Unemployed	155	41.9
Marital status	Married	191	51.6
	Unmarried	179	48.4
Disclosure status	Yes	302	81.6
	No	68	18.4
ART drug	NVP based	199	53.8
	EFV based	171	46.2
Clinical stage	I & II	66	17.8
	III & IV	302	82.2
Baseline CD4 count	<200 cells/mm^3^	221	59.7
	≥200 cells/mm^3^	149	40.3
*Median and IQR of CD4 count *			169 (IQR = 87.5–61.3)
*Mean and s.d of treatment duration *			47.7 (s.d = 26.3)

**Table 2:** Effect of selected sociodemographic, clinical, and other characteristics on self-reported adherence rate in Dubti Hospital, Afar, East Ethiopia, 2014.

Variables	Bivariate analysis	Multivariable analysis
	Crude OR (95% CI)	Adjust OR (95% CI)
Residence		
Rural	1.53 (0.82–2.85)^*^	
Urban	1.00	
Occupation		
Employed	1.48 (0.86–2.85)^*^	
Unemployed	1.00	
WHO clinical stage		
I & II	1.00	
III & IV	1.59 (0.75–3.38)^*^	
Baseline CD4 count		
<200 cells/mm^3^	0.71 (0.42–1.19)^*^	
≥200 cells/mm^3^	1.00	
Treatment duration		
1–6	1.00	1.00
7–12	0.14 (0.03–0.61)^*^	0.12 (0.028–0.54)^**^
13–24	0.199 (0.06–0.61)^*^	0.19 (0.06–0.578)^**^
25–36	0.254 (0.09–0.71)^*^	0.24 (0.086–0.68)^**^
>36	0.146 (0.06–0.35)^*^	0.14 (0.056–0.33)^**^

^*^The variables significantly associated with the outcome (*P* < 0.25) in bivariate analysis and ^**^the variables significantly associated with the outcome at 95% level of significant (*P* < 0.05) in multivariable logistic regression for adjusting all variables listed in the table.
